# Enhanced Thermoelectric Performance of Bi_2_O_2_Se with Ag Addition

**DOI:** 10.3390/ma8041568

**Published:** 2015-04-01

**Authors:** Bin Zhan, Yaochun Liu, Jinle Lan, Chengcheng Zeng, Yuan-Hua Lin, Ce-Wen Nan

**Affiliations:** 1 State Key Laboratory of New Ceramics and Fine Processing, School of Materials Science and Engineering, Tsinghua University, Beijing 100084, China; E-Mails: zhanb10@mails.tsinghua.edu.cn (B.Z.); ccc13@mails.tsinghua.edu.cn (C.Z.); cwnan@mail.tsinghua.edu.cn (C.-W.N.); 2 School of Materials Science and Engineering, University of Science and Technology Beijing, Beijing 100083, China; E-Mail: liuyaoch@126.com; 3 State Key Laboratory of Organic-Inorganic Composites, Beijing University of Chemical Technology, Beijing 100029, China; E-Mail: lanjl@mail.buct.edu.cn

**Keywords:** Bi_2_O_2_Se, thermoelectric, spark plasma sintering, Ag addition

## Abstract

Polycrystalline Bi_2_O_2_Se/Ag nanocomposites were synthesized by spark plasma sintering process. Their thermoelectric properties were evaluated from 300 to 673 K. With the addition of silver, the conductive second phase Ag_2_Se and Ag can be observed, which results in a significant enhancement of electrical conductivity. The maximum conductivity is 691.8 S cm^−1^ for Bi_2_O_2_Se/20 vol.% Ag, which increased nearly 500 higher times than the pure Bi_2_O_2_Se bulk. *ZT* value can be enhanced greatly, ~0.07, for Bi_2_O_2_Se/5 vol.% Ag at 673 K, which is two times larger than the pure sample.

## 1. Introduction

Thermoelectric materials and devices can realize direct conversion between heat and electricity that have attracted great interest owing to their widespread applications, such as solid-state cooling, power generation, and waste heat recovery [1,2]. The efficiency for energy conversion is characterized by a dimensionless figure of merit *ZT* = *S*^2^σ*T/*κ, where *T*, *S*, σ, and κ are the absolute temperature, Seebeck coefficient, electrical conductivity and thermal conductivity, respectively. An outstanding thermoelectric material requires a higher power factor (*PF* = *S*^2^σ) and a lower thermal conductivity.

Until now, alloys still exhibit the best thermoelectric performance, such as Bi_2_Te_3_, PbTe, SnSe, *etc.* [3,4,5], and show good practical prospects. In view of the low cost of raw materials and the high stability, thermoelectric oxides have been considered as promising candidates for high temperature applications. Typical oxides [1,6,7,8,9] (such as Ca_3_Co_4_O_9_, CaMnO_3_, and ZnO) have been extensively investigated over the past 20 years. However, their *ZT* values are still too low to be used in commercial applications due to the mediocre electrical conductivity and high thermal conductivity. Recently, a *p*-type oxide material, BiCuSeO, with a low intrinsic thermal conductivity has attracted great attention; its optimized *ZT* value can reach 1.4 at 923 K [10,11,12]. Therefore, we consider looking for a low thermal conductivity oxide for *n*-type thermoelectric applications.

As reported by *Ruleova et al.* [13], Bi_2_O_2_Se exhibits a very low thermal conductivity (0.7~0.75 Wm^−^^1^·K^−^^1^ at 800 K), combined with moderate power factor; hence it is expected to be a potential thermoelectric oxide. This compound is formed by partial replacement of selenium atoms with oxygen atoms in Bi_2_Se_3_, which belongs to so-called Sillen compounds and shows a (Na_0.25_Bi_0.75_)_2_O_2_Cl-type structure (D_4h_^17^). Similarly, Bi_2_O_2_Se exhibits a layered structure that is composed of tetragonal (BiO)*_n_* layers with Se occupying inter-layer positions, which result in the low thermal conductivity. Up to now, the reports about the thermoelectric performance of Bi_2_O_2_Se are still insufficient [14,15,16,17]. We have attempted to enhance the thermoelectric properties of Bi_2_O_2_Se by tetravalence Sn doping, or introducing Bi deficiencies. The Bi deficiencies did not significantly affect the electrical conductivity, but were in favor of orientation alignment of grains. The introduction of Sn brought about a high electrical conductivity and the highest *ZT* value can reach 0.20 at 773 K [18].

However, its electrical conductivity is still not enough (~60 S cm^−^^1^ at 773 K). In general, silver addition is used to enhance electrical conductivity and achieve the favorable results [19,20,21], thus it is considered to optimize the electrical conductivity of Bi_2_O_2_Se. In this work, we prepared the Bi_2_O_2_Se/Ag composites by spark plasma sintering (SPS), and evaluated their thermoelectric performances.

## 2. Results and Discussion

**Figure 1:**
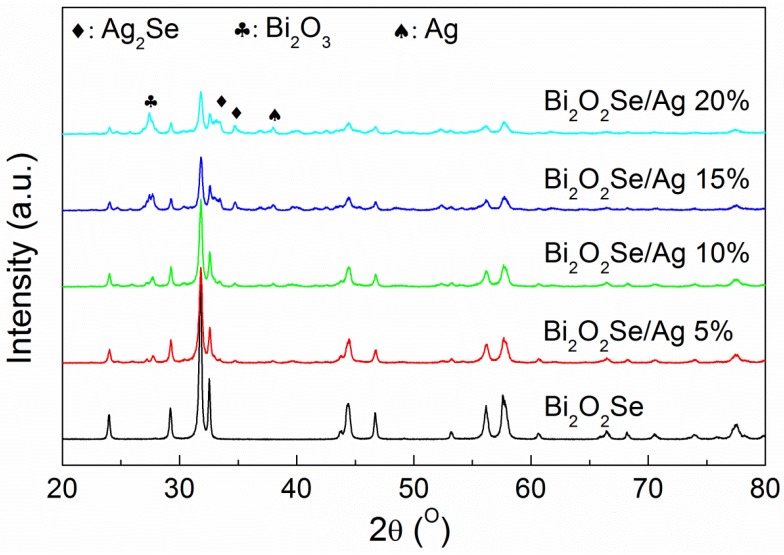
Room temperature X-ray diffraction patterns of Bi_2_O_2_Se/Ag composites.

Figure 1 shows the X-ray diffraction (XRD) patterns at room temperature of Bi_2_O_2_Se/Ag composites. The main phase corresponds to Bi_2_O_2_Se with a tetragonal structure in *I4/mmm* space group. As seen in Figure 1, the intensity of main phase in composites is decreased, while the second phases can be observed clearly as the Ag additive increasing. After matching the peaks, the second phases are composed of Ag, Ag_2_Se and Bi_2_O_3_. Extra silver is added as an independent additive, therefore the presence of an Ag phase is reasonable. Since Se exists in the form of a single atomic layer in the lattice, the bond between (BiO)*_n_* layers and Se layers should be weak; so it is possible to cause the chemical reaction between the silver and selenium. A reasonable assumption is that:

(1) $${\mathrm{Bi}}_{2}{\text{O}}_{2}\text{Se}+2\mathrm{Ag}+\frac{1}{2}{\text{O}}_{2}\to {\mathrm{Bi}}_{2}{\text{O}}_{3}+{\mathrm{Ag}}_{2}\mathrm{Se}$$

Obviously, more Ag additive will lead to the stronger second phase as the reaction proceeds. Meanwhile, the strength of the main phase decreases with the reaction of Bi_2_O_2_Se. This is consistent with the variation of peaks in Figure 1. That is to say, the introduction of the silver additive will undermine the stability of the Bi_2_O_2_Se structure, which is not an expected result.

**Figure 2:**
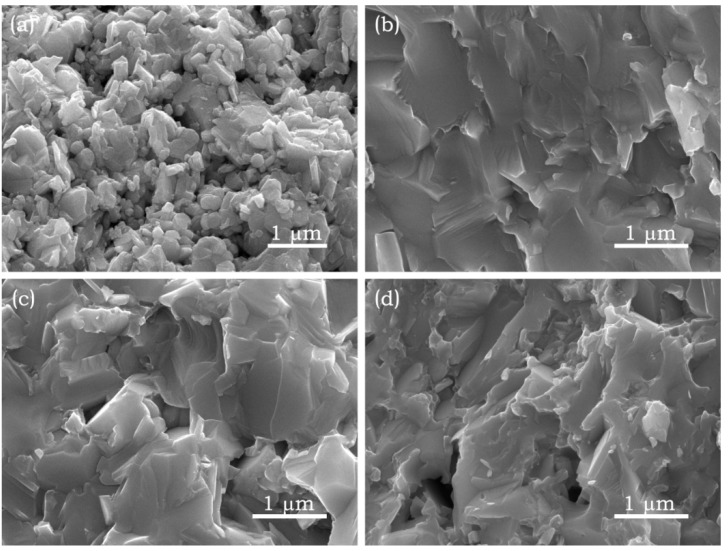
Field emission scanning electron microscopy of Bi_2_O_2_Se/Ag fracture surfaces, (**a**) pure sample; (**b**) 5 vol.% Ag; (**c**) 10 vol.% Ag; and (**d**) 20 vol.% Ag.

The fractured-surface microstructure in Bi_2_O_2_Se/Ag composites is presented in Figure 2. The pure sample shows a typical lamellar structure as seen in Figure 2a, and the thickness of flaked grains is around 200 nm. It exhibits a loose structure that shows a relative density of 84.5%. The microstructure obviously becomes more compact with the composite of silver, and the obtained experimental densities are 9.264, 9.170, 9.028, and 8.856 g·cm^−3^ for Bi_2_O_2_Se/Ag composites with volume ratios 5%, 10%, 15%, and 20%, respectively. As shown in Figure 2b, the grain size of sample significantly increases, and the binding between grains becomes closer compared with the pure sample. As we know, Bi_2_O_3_ is a common sintering aid; therefore it can notably improve the sintering performance of Bi_2_O_2_Se ceramics by Equation (1). However, the loose nanostructure has been destroyed, which will lead to a deterioration of thermal conductivity. With the increasing content of Ag, a similar change can be found; a small amount of pores were observed, which is consistent with the change of density being caused by the chemical reaction. Further, some nano-particles can be discovered in Figure 2d; they may originate from the precipitation of second phases, which can act as scattering centers for enhancing phonon scattering.

**Figure 3:**
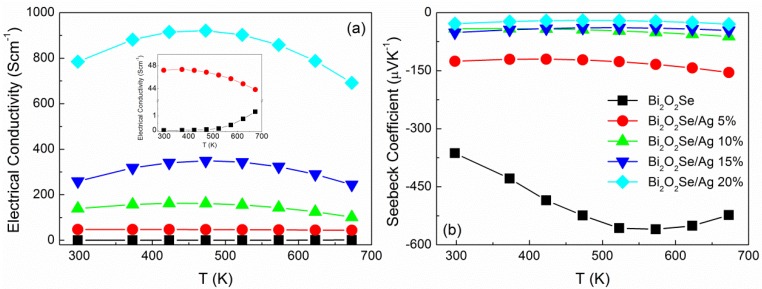
Temperature dependence of (**a**) electrical conductivity; and (**b**) Seebeck coefficient for Bi_2_O_2_Se/Ag composites.

The temperature dependence of electrical conductivity for Bi_2_O_2_Se/Ag composites from 300 to 673 K is shown in Figure 3a. A remarkable enhancement of electrical conductivity can be obtained by Ag addition. Their values at 673 K can reach 1.29, 43.84, 101.55, 243.91, and 691.79 S·cm^−^^1^ for different volume ratio of Ag (0%–20%), respectively. The pure sample exhibits typical semiconductor behavior, in which electrical conductivity increases with the temperature rising. Since the introduction of silver, a similar variation can be found in Figure 3a. The electrical conductivity increased first and then decreased as the temperature increased. It is well known that Ag is an excellent conductor (~10^5^ S·cm^−^^1^), which has a negative temperature coefficient. This does not match with the obtained results, thus the change of electrical conductivity may be mainly controlled by conductive phase Ag_2_Se. As reported by Day [22], that the resistivity of Ag_2_Se decreased before 400 K and then increased with temperature after phase transition, the same variation in Bi_2_O_2_Se/Ag composites can be observed as seen in Figure 3a. Therefore, we can infer that the abnormal electrical conductivity stems from the effect of Ag_2_Se. With the introduction of silver, it is uniformly dispersed in the matrix; however, a small amount of Ag_2_Se formed by Equation (1) and was wrapped in large grains as shown in Figure 2b. Carrier transport between them is not smooth and the electrical conductivity exhibits a limited increase. As the volume ratio increases to 10%, more conductive phase will be formed, the carrier concentration and transport route can be optimized. Further increasing the content of Ag to 20%, excessive second phases precipitated in the form of nanoparticles; the electrical conductivity shows significant enhancement combined with high carrier concentration and good transport properties in composite.

Temperature dependence of Seebeck coefficient for Bi_2_O_2_Se/Ag composites is shown in Figure 3b. In the whole temperature range from 300 to 673 K, the Seebeck coefficients are all negative, indicating a *n*-type electrical conduction behavior. The pure sample shows a high Seebeck coefficient, which ranges from −363.3 to −559.9 μVK^−^^1^. This value can be obtained from the layered structure, which consists of insulating (BiO)*_n_* layers and the conductive Se layers stacked alternately, forming natural superlattices. The two dimensional carriers confinement in the natural superlattices in Bi_2_O_2_Se is similar to the structure of BiCuSeO system [23,24]. With the addition of Ag, the absolute Seebeck coefficients exhibit dramatic reduction, −154.8, −61.7, −46.3, and −29.5 μVK^−^^1^ for Bi_2_O_2_Se/Ag composites with volume ratios 5%, 10%, 15%, and 20%, respectively. As mentioned before [22], the Seebeck coefficient of Ag_2_Se is from −140 to −20 μVK^−^^1^, which is affected by the process conditions, and it also shows a turning point attributed to the phase transition. A similar phenomenon can be observed in composites by Ag addition. With the formation of Ag_2_Se, the carrier concentration increased, therefore a decrease of Seebeck coefficient can be obtained. Moreover, it can be found in Figure 3b that the variation of value is small when volume ratio of Ag is over 10%, which indicates the electrical conductivity is not only affected by carrier concentration.

Power factor (*PF*) is calculated from electrical conductivity and Seebeck coefficient as *S*^2^σ. The values of *PF* at 673 K are 0.353, 1.050, 0.387, 0.522, and 0.604 μW·cm^−^^1^·K^−^^2^ for different volume ratio of Ag (0%–20%), respectively. For high content of Ag, the power factor increases with the electrical conductivity increasing, even with a low Seebeck coefficient. The highest value of power factor can be obtained in a sample of 5 vol.% Ag, which is associated with the optimized electrical conductivity while maintaining a moderate Seebeck coefficient. This shows once again that thermoelectric performance is a comprehensive consideration, with just high electrical conductivity or high Seebeck coefficient, it is difficult to obtain high power factor. We usually need to compromise to get an optimum result.

**Figure 4:**
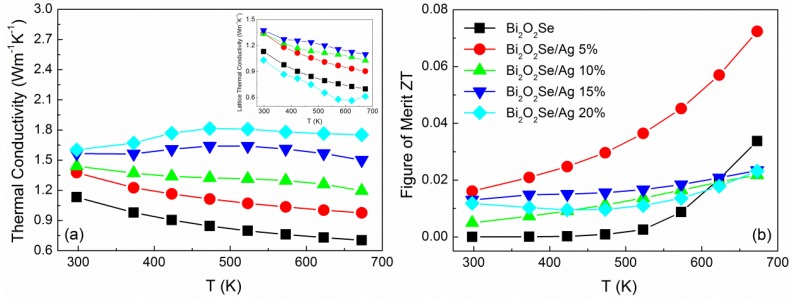
Temperature dependence of (**a**) thermal conductivity; and (**b**) *ZT* value for Bi_2_O_2_Se/Ag composites.

Temperature dependence of thermal conductivity (κ) between 300 and 673 K is shown in Figure 4a. The thermal conductivity at 673 K is 0.704, 0.976, 1.196, 1.501, 1.752 W·m^−1^·K^−1^ for Bi_2_O_2_Se/Ag composites with volume ratios 5%, 10%, 15%, and 20%, respectively. The huge increase in thermal conductivity may come from the presence of conductive phase Ag_2_Se, as in the reported result [22] of about 1.5~4 W·m^−1^·K^−1^. Especially in higher Ag content, the composites exhibit a similar feature to Ag_2_Se that thermal conductivity increases with temperature and then levels off after phase transition. As we know, thermal conductivity (κ) can be expressed by the sum of the lattice thermal conductivity (κ*_l_*) and electronic thermal conductivity (κ*_e_*) as κ = κ*_l_* + κ*_e_*. Usually, the electronic thermal conductivity can be calculated by Wiedemann-Franz’s law as below:

(2) $$\kappa_{e}=L\sigma T$$

where *L* is the Lorenz constant, σ is the electrical conductivity, and *T* is the absolute temperature. The calculated κ*_e_* in the entire temperature range is similar to the change of electrical conductivity, which shows a significant rise from 0.002 to 1.136 W·m^−1^·K^−1^. Based on the electronic thermal conductivity, the lattice thermal conductivity can be calculated. At lower content of silver, κ*_l_* increased with more Ag_2_Se formation and ceramics density improvement. When the vol.% is increased to 20%, the lattice thermal conductivity suddenly reduced to 0.616 W·m^−1^·K^−1^, which may derive from the precipitation of nanoparticles that can obviously improve on the scattering of phonons.

Based upon the electrical and thermal transport properties, the dimensionless figure of merit (*ZT*) is calculated as shown in Figure 4b. The highest *ZT* value reaches 0.072 at 673 K for Bi_2_O_2_Se/5 vol.% Ag, which demonstrates 115% improvement compared with pristine Bi_2_O_2_Se. With more Ag additive, the *ZT* values are lower than the pure sample at high temperature. This indicates the great improvement of electrical conductivity is insufficient to compensate for the loss of Seebeck coefficient and thermal conductivity. Meanwhile, the Ag addition will affect the stability of Bi_2_O_2_Se, therefore it may not be a good choice for the enhancement of thermoelectric performance of Bi_2_O_2_Se.

## 3. Experimental Section

Polycrystalline Bi_2_O_2_Se/Ag composites were prepared by a two-step method. First, the pure Bi_2_O_2_Se powders were synthesized by a similar method as our previous report [18] using the commercial grade Bi_2_O_3_, Bi, and Se powders with high purity. Then, Bi_2_O_2_Se powders were mixed with silver powders by volume ratio from 0% to 20% in a high-energy planetary ball milling in vacuum for 6 h. Finally, the obtained powdered materials were sintered into bulk ceramics at 823 K for 5 min under a pressure of 50 MPa by spark plasma sintering (1050 T, Sumitomo Coal Mining Co. Ltd., Tokyo, Japan).

The X-ray diffraction (XRD) with a D/MAX-2550V diffractometer (Rigaku, Tokyo, Japan; Cu Kα radiation) and scanning electron microscopy (SEM, JSM-6460LV, JEOL, Tokyo, Japan) were used to investigate the phase composition and microstructure of Bi_2_O_2_Se/Ag samples, respectively. A rectangular shaped sample with the dimensions of 15 × 3 × 3 mm^3^ was used to measure the electrical conductivity and Seebeck coefficient with a computerized homemade apparatus, and the measurement was perpendicular to the pressure direction. The temperature dependence of electrical conductivity was measured from room temperature to 673 K by a four-probe method. Seebeck coefficient was obtained from the slope of the linear relation between Δ*V* and Δ*T*, where Δ*V* is the thermoelectromotive force produced by the temperature gradient Δ*T*. The thermal conductivity κ was determined by the thermal diffusivity (α), the heat capacity (*C_p_*), and the density (ρ), in accordance with the relationship κ = α*·C_p_·*ρ, where laser flash apparatus Netzsch LFA 457 (Selb, Germany) was used to measure the thermal diffusivity and specific heat in the thickness direction of a square-shaped sample whose dimensions are 10 × 10 mm^2^ and ~1 mm in thickness, which is parallel to the pressure direction. The relative bulk density was measured by the Archimedes method. The estimated measurement accuracies are listed below: 5% for the electrical resistivity, 10% for the Seebeck coefficient, and 15% for the thermal conductivity.

## 4. Conclusions

In summary, *n*-type Bi_2_O_2_Se/Ag nanocomposite thermoelectric materials have been synthesized by a solidstate reaction followed with spark plasma sintering process. Thermoelectric properties were investigated in the temperature range from 300 to 673 K. Our results show that the introduction of Ag additive is an effective way to obtain high electrical conductivity, which can rise from 1.3 to 691.8 S·cm^−^^1^ at 673 K. However, silver will cause the dislocation of selenium and the formation of Ag_2_Se, leading to a low Seebeck coefficient and high thermal conductivity. The highest *ZT* value of 0.072 was obtained at 673 K for Bi_2_O_2_Se/5 vol.% Ag.
